# Tetrahydrocurcumin (THC) enhanced the clearance of *Cryptococcus deneoformans* during infection in vivo

**DOI:** 10.1007/s10482-023-01830-3

**Published:** 2023-04-25

**Authors:** Tianli He, Zhiran Jin, Wei Hu, Xiaoxue Xia, Donghui Li, Weiyun Yao, Guangnan Li, Xuefeng Zhou, Guoqiang Song

**Affiliations:** 1 Department of Radiotherapy, Changxing People’s Hospital, No. 66, Taihu Road, Changxing, Huzhou, 313100 Zhejiang China; 2 Department of Surgery, Changxing People’s Hospital, No. 66, Taihu Road, Changxing, Huzhou, 313100 Zhejiang China; 3 Department of Infectious Diseases Department of Respiratory, Changxing People’s Hospital, No. 66, Taihu Road, Changxing, Huzhou , 313100 Zhejiang China; 4 Department of Neurology, Changxing People’s Hospital, No. 66, Taihu Road, Changxing, Huzhou, 313100 Zhejiang China; 5 Department of Respiratory Medicine, Changxing County Hospital of Traditional Chinese Medicine, Huzhou, 313100 Zhejiang China; 6 Department of Respiratory Medicine, Changxing People’s Hospital, No. 66, Taihu Road, Changxing, Huzhou, 313100 Zhejiang China

**Keywords:** Tetrahydrocurcumin (THC), *Cryptococcus deneoformans*, Tight junctions, Claudin-4

## Abstract

Cryptococcal species often cause lung infections and are the main cause of fungal meningitis. Claudin-4 appears to be a major structural component that maintains a tight alveolar barrier and prevents fluid and electrolyte leakage into the alveolar space. We aimed to determine whether S7-tetrahydrocurcumin (THC) could clearance of *C. deneoformans* and regulate claudin-4 expression during *C. deneoformans* infection. We investigated the effect of THC on *C. deneoformans* infection and its possible mechanism in vivo. Transmission electron microscopy was used to observe the ultrastructure of the lung tissue and the invasion of *Cryptococcus*. To clarify the effect of THC, we examined claudin-4, c-Jun, and Smad2 expression. We also measured claudin-4 expression in pulmonary specimens from clinical patients. THC reduced cryptococcal cell invasion in the lungs, improved alveolar exudation, and reduced inflammation. Pretreatment with THC suppressed c-Jun and Smad2 expression, resulting in significantly increased claudin-4 levels. In contrast, the expression of claudin-4 in clinical specimens from patients with cryptococcal infection was higher than that in normal specimens. THC enhanced the clearance of *C. deneoformans* during infection in vivo. We investigated the expression of claudin-4 and the possible mechanism of THC against *C. deneoformans* infection.

## Introduction

*Cryptococcus deneoformans* is the leading cause of fungal meningitis in populations with impaired immunity (Kwon-Chung 2014). *Cryptococcus* often causes lung and brain infections in individuals who are immunocompromised (Taylor-Smith 2017). The global burden of this disease is estimated to be close to 1 million cases, with 700,000 deaths annually (Park 2007).

The rising prevalence of various diseases is believed to be closely linked to, or even dependent upon, modifications occurring within the tight junctions (Cereijido 2007). Claudins, which are major transmembrane proteins that localize at tight junctions, were first discovered by Furuse et al. in the chicken liver in 1998 (Furuse 9). Previous studies suggest that claudins 1, 3, 4, 5, 7, 10, and 18 (splice variant 1) are expressed in bronchi and bronchioles, and some have also been detected in the distal lung (Coyne 2003). Claudins 3, 4, and 7 are predominantly expressed in alveolar type II cells. Claudin-4 appears to be a major structural component that maintains a tight alveolar barrier and prevents the leakage of fluid and electrolytes into the alveolar space. Wray C and colleagues demonstrated that claudin-4 suppressed air space fluid clearance and exacerbated ventilator-induced pulmonary oedema in vitro (Wray 2009). Consistent with this finding, the expression of claudin-4 is associated with alveolar fluid clearance (Rokkam 2011). Amasheh et al. showed that the downregulation of claudin-1 and upregulation of claudin-2 was mediated by the TNF-/NF-κB pathway in the intestinal HT-29/B6 cell line. These effects could be suppressed by the herbal compound berberine (Amasheh 2010).

Smad proteins, distinguished as members of the TGF-β family due to their structural and functional characteristics in signaling, have been implicated in hepatic metastasis in colorectal cancer (Halder 2008). C-Jun, a key component of the protein activator protein-1 (AP-1) complex, plays a significant role in numerous cellular processes, including proliferation, apoptosis, survival, tumorigenesis, and histomorphogenesis. Despite years of rigorous research, the intricate functional properties of c-Jun, positioned at the core of the molecular network, remain to be fully unravelled (Meng 2011). Christina Van Itallie and colleagues observed that claudin-4 overexpression led to a reduction in paracellular electrical conductance and impacted paracellular ion selectivity (Itallie 2001). In another study, Patrick Michl and his team demonstrated that claudin-4 could suppress invasiveness and the metastatic phenotype of pancreatic cancer cells through the TGF-β and Ras signaling pathways (Michl 2003). Furthermore, Michl's team revealed claudin-4 overexpression in pancreatic cancer and illustrated that targeting claudin-4-expressing tumors with CPE resulted in extensive tumor cell necrosis and a substantial decrease in tumor growth (Michl 2001). Notably, claudin-4 is also found at elevated levels in prostate tissue (Long 2001). The overexpression of both claudin-3 and claudin-4 has been linked to malignancy in ovarian cystadenoma (Rangel 2003).

Curcumin improves intestinal barrier function through the modulation of intracellular signalling and the organization of tight junctions (Wang 2017). Erin E. Olson et al showed that S7-tetrahydrocurcumin (THC), which is derived from extracts rich in curcuminoids and catechins could reduce immune cell inflammation and preserve tight junction integrity in an in vitro coculture model of intestinal inflammation (Olson 2020). Interestingly, THC could alter these functional and molecular changes (Mondal 2019).

Based on these findings, the present study was conducted to examine the effect of THC, a major secondary metabolite of curcumin, and the possible mechanism against *C. deneoformans* infection.

## Materials and methods

### Materials

THC was obtained from China Haoxin Biotechnology Co., Ltd. and subsequently dissolved in dimethyl sulfoxide (DMSO) before being stored as a frozen 200 µM stock solution. The following primary antibodies were used: claudin-4 (Abcam, ab15104), c-Jun (Abcam, ab40766), Smad2 (Abcam, ab63576), and β-actin (1:1000 Cell Signaling Technology, CST). The secondary antibody was goat anti-mouse/rabbit IgG (1:5000 Cell Signaling Technology, CST).

### C. deneoformans

The *C. deneoformans* strain 52D (ATCC 24067) was obtained from the American Type Culture Collection (Manassas, VA). For infection, yeast were grown to stationary phase after an incubation period of 48 to 72 h at 37 °C in Sabouraud dextrose broth (1% neopeptone and 2% dextrose; Difco, Detroit, MI) on a shaker. Cells were washed twice with saline, counted on a haemocytometer, and diluted to 3.3 * 10^5^ CFU/ml in nonpyrogenic saline.

### Mouse controls

Male KM mice (5–6 weeks of age) were obtained from Shanghai SLAC Laboratory Animal Co., Ltd. The mice were randomly assigned to five groups: (1) the normal group (n = 6); (2) the control group: *C. deneoformans* plus DMSO (n = 8); (3) the low-dose THC group: *C. deneoformans* plus 50 mg/kg/d THC (n = 8); (4) the middle-dose THC group: *C. deneoformans* plus 100 mg/kg/d THC (n = 8); and (5) the high-dose THC group: *C. deneoformans* plus 200 mg/kg/d THC (n = 8). THC was administered to mice by gavage for one week. Mice were infected with *C. deneoformans* on day 8. All experiments were approved by the Ethics Committee of Changxing People’s Hospital.

### Intratracheal inoculation of *C. deneoformans*

Mice were anesthetized using intraperitoneal injections of pentobarbital sodium. Fifty microlitres of *C. deneoformans* (10^5^ CFU) was dripped into the trachea through a syringe. Body weight was measured every 4 days for 5 weeks, and then the mice were sacrificed by intraperitoneal injections of pentobarbital sodium.

### Histological analysis

Three specimens were randomly selected from each group. Lung tissues were fixed in 4% formaldehyde for 3–5 days. After paraffin embedding, 4 µm sections were cut and stained with haematoxylin and eosin. Sections were analysed using light microscopy.

### Immunohistochemical staining

The lung tissues were fixed in 4% formaldehyde, embedded in paraffin, and cut into 4-µm-thick sections. Before being boiled in citrate buffer, the slides were immersed in 3% hydrogen peroxide for 15 min to block endogenous peroxidase activity. The slides were incubated with claudin-4, c-Jun, and Smad2 antibodies and then incubated with HRP-conjugated antibodies. The slides were visualized with a DAB Horseradish Peroxidase Colour Development Kit (Beyotime) and counterstained with haematoxylin. The images were analysed by Image-Pro Plus 6.0 software.

### Visualization of the interaction of *C. deneoformans* with the lung by TEM

Lung tissues were fixed with 2.5% glutaraldehyde phosphate buffer. Then, the samples were postfixed in osmium acid for 1 h, stained with 2% uranyl acetate in an aqueous solution for 30 min, dehydrated through a graded alcohol series, and embedded in Spurr resin. The tissues were cut into 120 nm sections, poststained in uranyl acetate and lead citrate, and viewed with a Philips TECNA-10 transmission electron microscope.

### Quantitative real-time PCR

Total RNA was extracted using a high-purity total RNA rapid extraction kit, and cDNA was synthesized using a PrimeScript™ RT reagent kit and HiScript-IIQ RT SuperMix (Vazyme, Piscataway, USA). The reaction was mixed in an ice bath and then reacted at 50 °C for 15 min and then at 85 °C for 2 min. The reaction was then stopped and stored at − 20 °C until use. Each sample was run in a final volume of 20.0 µl containing 10 µl of 2X ChamQ SYBR Colour qPCR Master Mix, 0.6 µl of forward primer (10 µM), 0.6 µl of reverse primer (10 µM), and 8.8 µl of template cDNA(claudin-4 forward, CCGCACTGTCTTGCTAATG and reverse, CAGAGGGGCCAACTCAA; c-Jun forward, GAACTCGGACCTTCTCACG and reverse, TGGGGCACAAGAACTGG; Smad2 forward, CCTTCCATGCGTCACAG and reverse, CGCACTCCCCTTCCTAT). β-actin served as the reference gene, and the 2-ΔΔCq technique was employed to ascertain the relative expression levels of each gene. Three samples were randomly chosen from each group to ensure a representative analysis.

### Western blot analysis

Three samples were randomly selected from each group for analysis. Lung tissues were lysed using RIPA buffer, and protein concentrations were determined employing a BCA kit from Beyotime. Subsequently, 30 μg of protein was isolated for further examination. Equal quantities of proteins were separated using either 4% or 12% SDS-polyacrylamide gels and then transferred onto nitrocellulose membranes provided by Millipore. The membranes were blocked using 5% nonfat milk in TBST (Tris-buffered saline, pH 7.4, with 0.05% Tween 20) and incubated with Claudin-4 antibody (1:100), c-Jun antibody (1:100), and Smad2 antibody (1:100)—all sourced from Abcam—overnight at 4 °C. Following this, the membranes were incubated with alkaline phosphatase-conjugated goat anti-mouse IgG or goat anti-rabbit IgG (1:5000) for 1 hour at room temperature. Finally, the signals were detected using an enhanced chemiluminescence detection kit to reveal the desired outcomes.

### Statistical analysis

The data were meticulously analyzed using GraphPad Prism version 9.0 (Graph Pad Software, San Diego, CA, USA) and are presented as the mean ± standard deviation (SD). To assess differences between groups, ANOVA was employed, followed by a Bonferroni multiple comparisons posttest. A *P* value of < 0.05 was deemed indicative of statistical significance. Each experiment was performed in triplicate to ensure consistency and reliability in the findings.

## Results

**Fig. 1:**
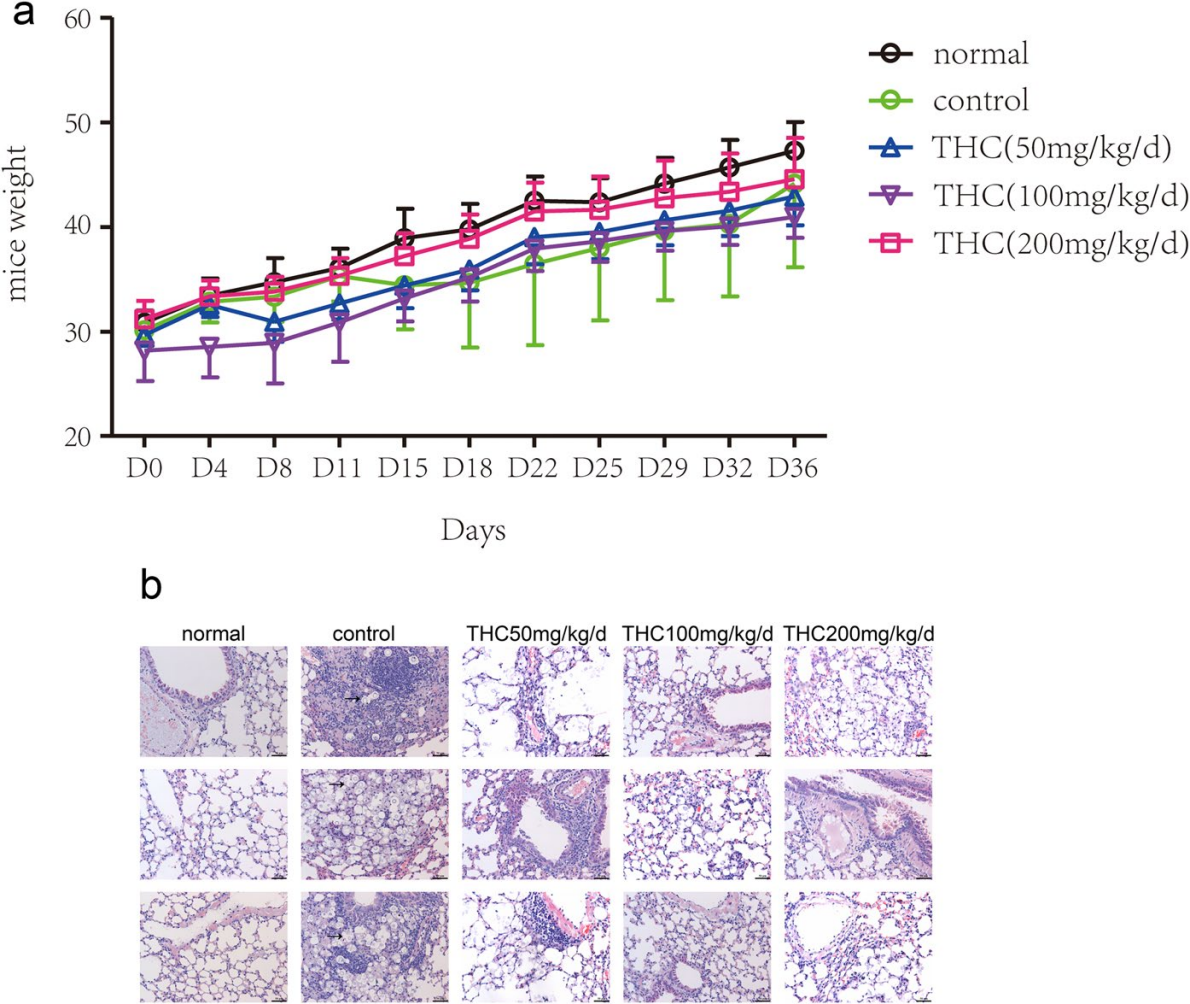
**a** Measurement of mouse weight every four days. **b** Histopathology of lung for Male KM mice infected with *C. deneoformans.* Three specimens were randomly selected from each group. Black arrowheads indicates C.neoformans cells stained hematoxylin and eosin. Many yeast cells appear as degenerated cells within macrophages

In order to investigate the impact of THC on Cryptococcus infection in mice, several treatment groups were established. The lung tissues from mice in each group were subsequently analyzed for further experimentation. Following cryptococcal infection, the other groups did not exhibit weight loss in comparison to the uninfected control group of mice (Figure 1a). Histological analysis showed that compared with those in the normal group, multiple granulomatous nodules appeared in the control group, and *Cryptococcus* was surrounded by large numbers of macrophages, lymphocytes, and a small number of neutrophils. Additionally, mild bronchial pneumonia, intra-alveolar haemorrhage, alveolar fusion, and interstitial widening were observed (Figure 1b). *Cryptococcus* was observed in one specimen in the THC low-dose group. No *Cryptococcus* was found in the other two specimens. We also observed mild bronchopneumonia, a small amount of haemorrhage in the alveoli, mild fusion of the alveoli, and widening of the interstitium. Additionally, these groups showed less severe inflammation than the control group. We observed a small amount of *Cryptococcus* in the middle-dose THC group. However, there was no obvious inflammation in the lungs, and the alveoli were normal. The results from the high-dose group were in between. In this experiment, THC reduced the invasion of cryptococcal cells in the lungs, improved alveolar exudation, and reduced inflammation.

**Fig. 2:**
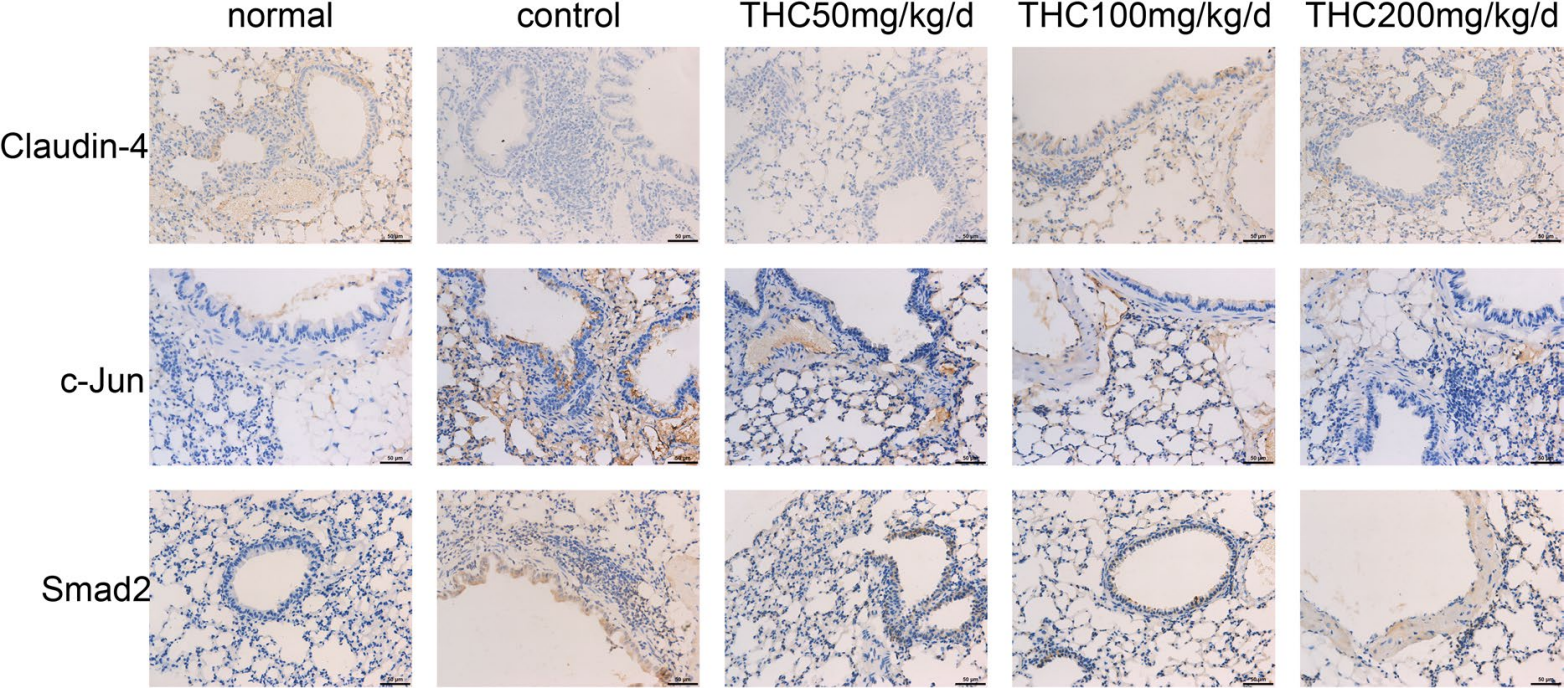
Immunohistochemical staining. **a** THC promoted claudin-4 expression and suppressed c-Jun and Smad2 expression compared to that in the control group. Compared with that in the control group, the expression of Claudin-4 in the presence of THC increased

**Fig. 3:**
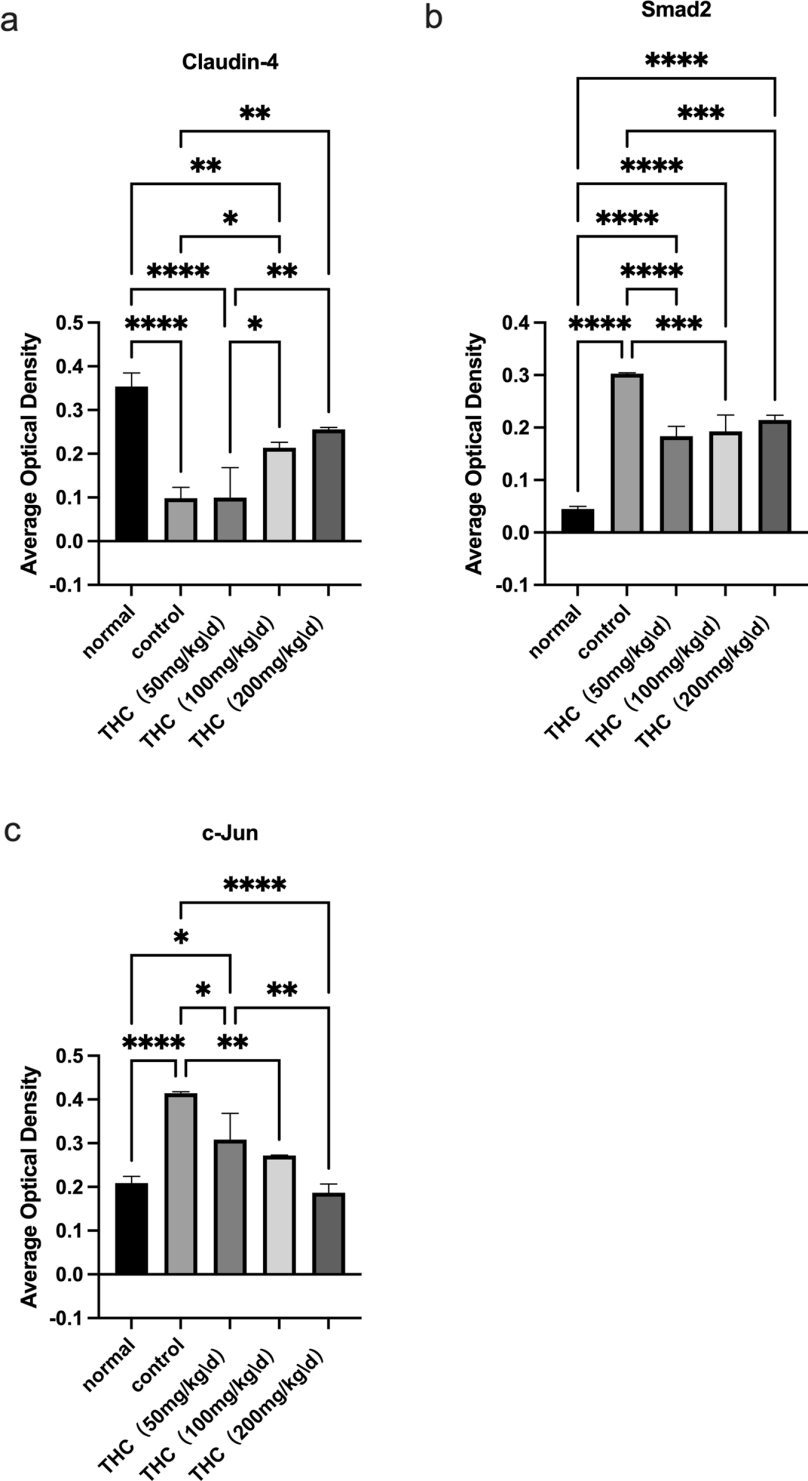
Averagel optical densities (AODs) of the various experimental groups on the end of the treatment period. (*p < 0.05, **p < 0.01, ***p < 0.001,****p < 0.0001)

We performed immunohistochemistry and found that THC promoted claudin-4 expression and suppressed c-Jun and Smad2 compared to those in the control group (Figure 2). Compared with the normal group, the expression of Claudin-4 in the control group was significantly reduced. Compared with claudin-4 expression in the control group, the expression of Claudin-4 in the presence of THC increased to varying degrees in each other group (Figure 3a). As shown in Figure 3b, the expression of Smad2 in the control group was significantly increased. Compared with the control group, the expression of Smad2 in each group in the presence of THC was reduced. The same effect on the expression of c-Jun was also observed (Figure 3c).

**Fig. 4:**
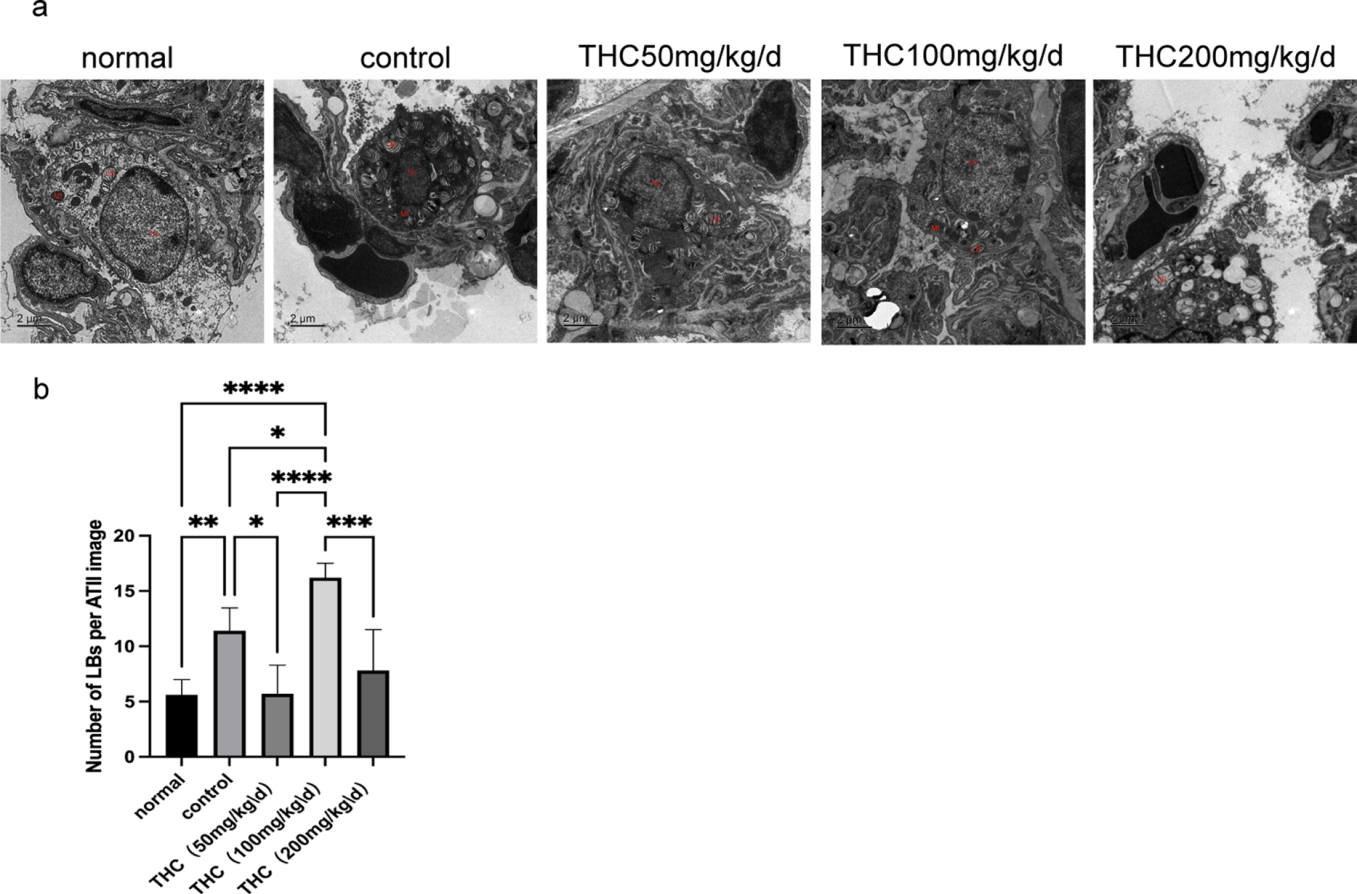
Electron microscopy of lung tissue from mice inoculated with *C. deneoformans*. **a** The ultrastructure of lung tissue and the invasion of *Cryptococcus* after treatment with THC.One sample were randomly selected from each group. The overview in a shows a well-preserved type II cell from a rat lung. Lamellar bodies (LB), nucleus (Nu) and mitochondria (Mt) are well preserved.Bars = 2 μm. **b** Analysis of LBs number within the alveolar epithelial type II cells( ATII).Values are expressed as mean ± SEM. (*p < 0.05, **p < 0.01, ***p < 0.001,****p < 0.0001), n = 5/group

Type I and type II alveolar epithelial cells had a complete structure, and the organelles were not damaged, but the lamellar bodies of type II alveolar epithelial cells were reduced or even vacuolated, and some capillaries were congested and blocked in the normal group (Figure 4a). In the control group, we observed a large number of lamellar bodies and capillary congestion and blockage. In the low-dose THC group, the number of lamellar bodies was in between. Capillary congestion was improved, but some red blood cells were exuded. Compared with the corresponding data in the THC low-dose group, the number of lamellar bodies was greater in the middle-dose THC group, and capillary congestion was improved, but some red blood cells were still exuded. The ultrastructure of the high-dose THC group was similar to that of the control group (Figure 4b).

**Fig. 5:**
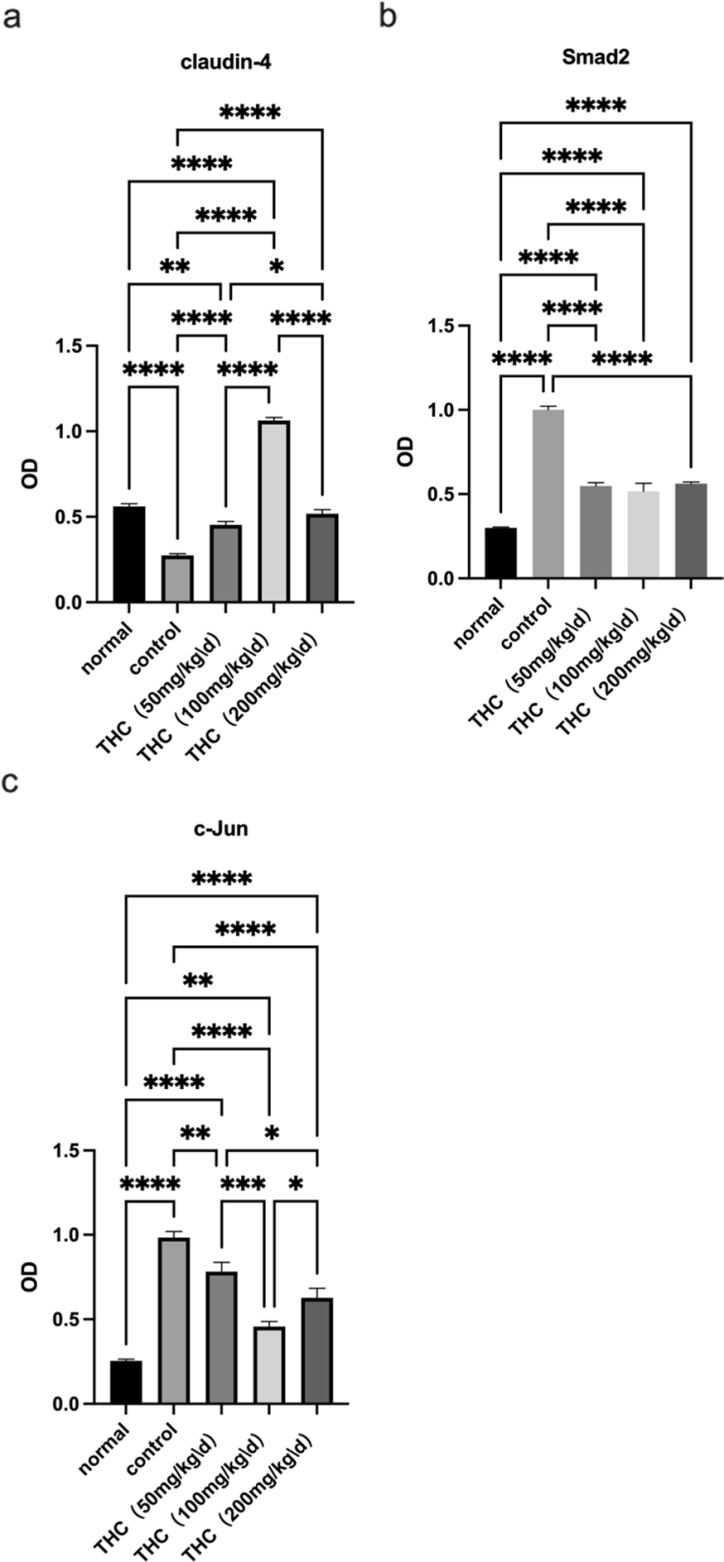
**a** THC upregulated the expression of claudin-4 compared with that in the control group. **b**, **c** Quantitative real-time PCR indicated that THC decreased the expression of c-Jun and Smad2 (*p < 0.05, **p < 0.01, ***p < 0.001). n = 3/group

Quantitative real-time PCR indicated that THC decreased the expression of c-Jun and Smad2 (Figure 5b and Figure 5c). However, THC upregulated the expression of claudin-4 compared with that in the control group (Figure 5a). Additionally, the middle-dose THC group showed the greatest effect.

**Fig. 6:**
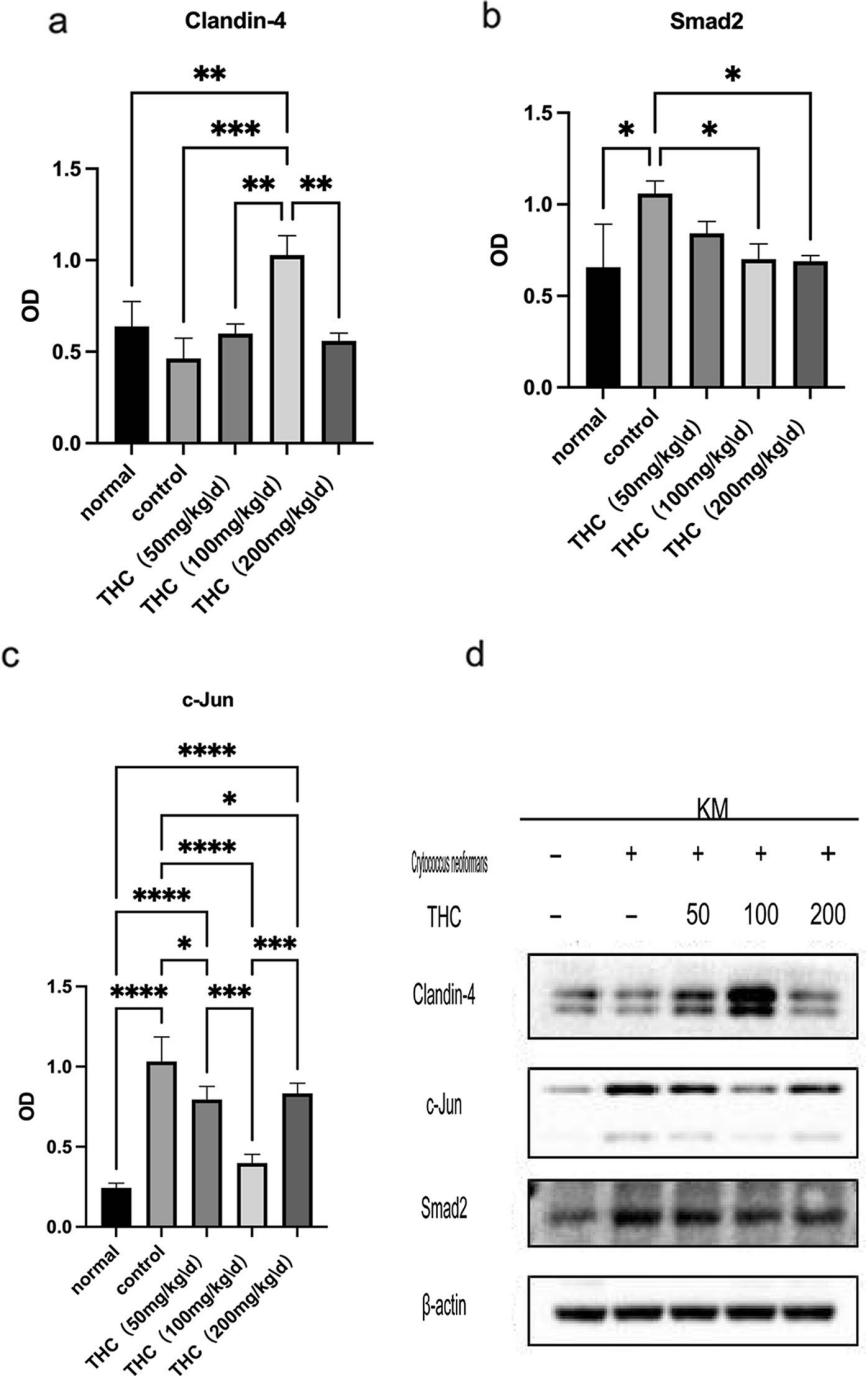
Western blotting indicated that claudin-4 was upregulated in the presence of THC in the lung tissues of mice. In contrast, the expression of c-Jun and Smad2 was suppressed by THC (*p < 0.05, **p < 0.01, ***p < 0.001). n = 3/group

Western blotting indicated that claudin-4 was upregulated in the presence of THC in the lung tissues of mice (Figure 6a). In contrast, the expression of c-Jun and Smad2 was suppressed by THC (Figure 6b, c). We also found that the effect was more notable in the group treated with 100 mg/kg THC (Figure 6d).

**Fig. 7:**
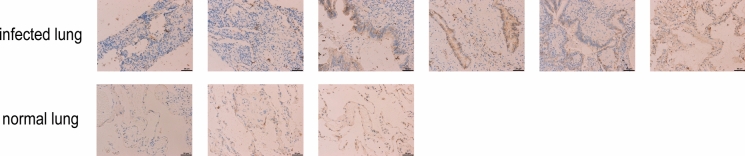
The expression of claudin-4 in pulmonary specimens from clinical patients

We also measured the expression of claudin-4 in pulmonary specimens from clinical patients (Table1). The expression of claudin-4 in lung tissues from clinical patients who were infected with *C. deneoformans* was significantly increased compared with that in normal lung tissues. (Figure 7). This finding was the opposite of what was observed in mice.

**Table 1:** The information about clinical specimens

Infected lung		Normal lung	
Age	Gender	Age	Gender
50	Male	40	Male
45	Male	58	Male
66	Femal	60	Femal
70	Male		
56	Femal		
48	Femal

## Discussion

Claudin-3, claudin-4, and claudin-18 are the predominant claudins expressed by the alveolar epithelium (Koval 2013). Many studies suggest that several human lung disorders are linked to claudin dysregulation. Charlie Wray et al demonstrated that increased claudin-4 expression early in acute lung injury represents a mechanism to limit pulmonary oedema that is dependent on PKC activation and the JNK MAPK pathway. Low expression of claudin-4 results in leakier tight junctions, decreased alveolar fluid clearance, and increased pulmonary oedema with mechanical ventilation (Wray 2009). However, the expression of alveolar epithelial claudin-4 is downregulated in sepsis, which increases the severity of lung injury (Cohen 2010). LSH Chuang et al observed that TGF-β signalling resulted in the dissolution of tight junctions (Chuang 2010). Chang et al also found that the cooperation of Runx3 and the TGF-β effector Smad directly upregulates claudin-1 transcription (Chang 2010).

Cryptococcus heteromorphous, an opportunistic fungal pathogen, infects the lungs via airborne transmission and frequently results in fatal meningoencephalitis. It has been reported that Cldn-4 functions as a paracellular sodium barrier and plays a somewhat ambiguous role in safeguarding the lung from acute injury, even though its contribution to normal lung physiology is minimal (Kage 2014). Ko Sato and colleagues discovered that there was no significant difference in pulmonary fungal load between Cldn-4+/+ and Cldn-4−/− mice at day 14 post-infection. Fungal clearance appeared normal in Cldn-4-deficient mice (Sato 2021).

In this study, we investigated the effect of THC and its possible mechanism against *C. deneoformans* infection in vivo. THC reduced the invasion of cryptococcal cells in the lungs, improved alveolar exudation, and reduced inflammation. Pretreatment with THC suppressed the expression of c-Jun and Smad2, resulting in a significant increase in claudin-4. In contrast, we found that the expression of claudin-4 was higher in clinical specimens from patients with cryptococcal infection than in normal specimens. This difference may be because the inflammatory response in the human body is more complicated than that in mice.

The study of Girish Rachakonda et al provided convincing evidence that TGF-β induced Claudin-4 expression through c-Jun signalling in non-small-cell lung cancer. Treatment with curcumin, a c-Jun inhibitor, abrogated TGF-β-induced claudin-4 expression, suggesting the involvement of the c-Jun pathway (Rachakonda 2016). Similarly, we have provided evidence that THC altered the expression of claudin-4 possibly via the c-Jun signalling pathway. In addition, we observed that THC pretreatment regulated the expression of Smad2 in response to *C. deneoformans* infection. Consistent with these results, Chuang LSH et al observed that TGF-β signalling resulted in the dissolution of tight junctions (Chuang 2010). Chang et al also found that cooperation between Runx3 and the TGF-β effector Smad directly upregulated claudin-1 transcription (Chang 2010).

THC possesses antioxidant, antifungal, and antimycotoxic properties; however, its typically low water solubility considerably restricts its potential application in water-based formulations (Coma 2011). Anne Loron et al.’s study revealed that, in preliminary tests, 10 µM THC did not inhibit the growth of F. graminearum. THC-loaded particles have demonstrated promising antifungal properties. Since the product does not interfere with fungal growth at this concentration, THC’s impact on toxin production can be assessed accordingly (Coma 2021). Shan Chen discovered that neither free nor encapsulated THC affected non-cancerous mouse embryonic cells, suggesting that THC is biocompatible with healthy non-melanoma cells and exhibits no biotoxicity (Shan 2021). THC's influence on host response has not been thoroughly validated. Perhaps from a more critical standpoint, the untreated THC treatment group’s effect should be examined. Evaluating THC’s impact on Cryptococcus activity in vitro is also essential. If THC has antifungal activity, it could potentially result from a weakened host response by reducing the fungal population. However, this hypothesis was not fully considered and verified in our experiments. In BALB/c mice infected with Cryptococcal strain 52D, Aditya V. Jain observed that strain 52D proliferated rapidly (1000-fold increase compared to the original inoculum) at week 1, but its subsequent growth was inhibited (weeks 2 and 3), and significant clearance was observed at week 4 compared to the peak level at week (Jain 2009). It is also necessary to expand the assay's time points in the experiment to thoroughly comprehend Cryptococcus clearance and the changes in the indicators over time. In clinical practice, patients are often infected with cryptococci first and then follow up with treatment. Therefore, a mouse model of cryptococcal infection can be considered in the laboratory before subsequent THC treatment.

This study is the first to demonstrate that THC can be used to address *Cryptococcus* pneumoniae. These findings suggest specific roles for claudin-4, c-Jun, and Smad2 during *C. deneoformans* infection, but the exact mechanism remains to be elucidated. Therefore, this report demonstrates for the first time that THC may be a potent therapeutic agent for cryptococcal pneumonia, but this finding must be tested further.
